# Clinical, humanistic, and economic burden of chronic obstructive pulmonary disease (COPD) in Canada: a systematic review

**DOI:** 10.1186/s13104-015-1427-y

**Published:** 2015-09-21

**Authors:** Tam Dang-Tan, Afisi Ismaila, Shiyuan Zhang, Victoria Zarotsky, Mark Bernauer

**Affiliations:** GlaxoSmithKline, 7333 Mississauga Road, Mississauga, ON L5N 6L4 Canada; GlaxoSmithKline, Research Triangle Park, NC USA; Clinical Epidemiology and Biostatistics, McMaster University, Hamilton, ON Canada; Optum, Eden Prairie, MN USA

**Keywords:** COPD, Chronic obstructive, Literature review, Burden, Burden of illness, Canada, Costs, Clinical, Economic, Humanistic, Quality of life

## Abstract

**Background:**

Chronic obstructive pulmonary disease (COPD) is a chronic, irreversible disease and a leading cause of worldwide morbidity and mortality. In Canada, COPD is the fourth leading cause of death. This systematic review was undertaken to update healthcare professionals and decision makers regarding the recent clinical, humanistic and economic burden evidence in Canada.

**Methods:**

A systematic literature search was conducted in PubMed, EMBASE, and Cochrane databases to identify original research published January 2000 through December 2012 on the burden of COPD in Canada. Each search was conducted using controlled vocabulary and key words, with “COPD” as the main search concept and limited to Canadian studies, written in English and involving human subjects. Selected studies included randomized controlled trials, observational studies and systematic reviews/meta-analyses that reported healthcare resource utilization, quality of life and/or healthcare costs.

**Results:**

Of the 972 articles identified through the literature searches, 70 studies were included in this review. These studies were determined to have an overall good quality based on the quality assessment. COPD patients were found to average 0–4 annual emergency department visits, 0.3–1.5 annual hospital visits, and 0.7–5 annual physician visits. Self-care management was found to lessen the overall risk of emergency department (ED) visits, hospitalization and unscheduled physician visits. Additionally, integrated care decreased the mean number of hospitalizations and telephone support reduced the number of annual physician visits. Overall, 60–68 % of COPD patients were found to be inactive and 60–72 % reported activity restriction. Pain was found to negatively correlate with physical activity while breathing difficulties resulted in an inability to leave home and reduced the ability to handle activities of daily living. Evidence indicated that treating COPD improved patients’ overall quality of life. The average total cost per patient ranged between CAN $2444–4391 from a patient perspective to CAN $3910–6693 from a societal perspective. Furthermore, evidence indicated that COPD exacerbations lead to higher costs.

**Conclusions:**

The clinical, humanistic and economic burden of COPD in Canada is substantial. Use of self-care management programs, telephone support, and integrated care may reduce the overall burden to Canadian patients and society.

## Background

Chronic obstructive pulmonary disease (COPD) is a persistent, irreversible, progressive disease exacting a heavy toll on patients and caregivers and is a leading cause of morbidity and mortality worldwide [1–4]. Estimates indicate that more than 10 % of the adult population are affected by COPD, and one in four adults over the age of 35 will develop COPD in their lifetime [5, 6]. In Canada, COPD is project to be the fourth leading cause of death behind heart disease, cancer and stroke and is expected to be the third leading cause of death by 2020 [3]. Exposure to environmental factors is thought to be the major underlying cause of COPD, with smoking being the most important risk factor [7–9]. Comorbidities, such as cardiovascular disease, are very common and are thought to contribute to the vast majority of COPD deaths [10–12].

The unique features of the Canadian universal healthcare system provide different challenges for government and health care providers alike in the delivery and implementation of health services. With the substantial burden and societal importance of COPD, it is important for Canadian healthcare professionals and decision makers to remain up to date with evidence of managing and treating COPD. A sizeable body of research on the burden of COPD in Canada has been conducted in recent years; however, a systematic review of recent evidence is lacking. The overall purpose of this systematic review is to update the knowledge of the burden of COPD in Canada by summarizing the most current, evidence-based information. The specific objective is to summarize the recent literature describing the clinical, humanistic and economic burden of COPD among Canadians.

## Methods

### Literature search

We conducted a search of the PubMed, EMBASE, and Cochrane databases to identify original research (observational and interventional studies, burden of illness studies, and cost of illness studies) published January 2000 through December 2012 on the burden of COPD in Canada. Non-systematic review articles, letters, editorials, commentaries, studies reporting summaries of meeting proceedings or conferences, abstracts or posters presented at scientific meetings, and studies examining the efficacy or effectiveness of specific pharmacotherapy interventions were not included. Each search was conducted using controlled vocabulary and key words and was limited to articles published in English, studies conducted with Canadian data, and studies involving humans. Additional articles were identified and added to each review through a review of the bibliographies of included articles and if identified in the other literature search (i.e. article with economic data found in humanistic literature search).

### Study selection

Titles and abstracts of articles identified were carefully screened in the initial review for relevance to the topic by a single reviewer. Articles were selected for inclusion based on predefined acceptance criteria, which included relevant patient population (i.e., adults/children diagnosed with COPD), study design [randomized controlled trial (RCT), observational study, systematic review/meta-analyses] and outcome measures (healthcare resource utilization, quality of life, healthcare costs). Complete articles were obtained for any article that categorized as ‘included’ or ‘unsure’ after the title and abstract review. All ‘unsure’ articles were then reviewed to make a final determination of inclusion or exclusion. A second, independent reviewer performed a check on a random sample of 20 % of the articles with discrepancies resolved through consensus. Articles identified as potentially relevant were obtained in full text for further evaluation.

### Data abstraction

Data abstraction forms were designed a priori. For articles that met predefined inclusion/exclusion criteria, key outcomes were abstracted and tabulated in summary tables. Key outcomes extracted included: emergency department visits, hospitalization and office visits in the clinical burden literature; quality of life measures in the humanistic burden literature; patient and population costs in the economic burden literature. In the economic burden section, reported costs were inflated to 2012 Canadian dollars using the Consumer Price Index from Statistics Canada (http://www.statcan.gc.ca). A second, independent reviewer performed a check on a random sample of the data abstracted from 20 % of the articles.

### Quality assessment

Quality was assessed by using internationally recognized methodological checklists from the National Institute for Health and Care Excellence (NICE) Guidelines Manual for RCT [13], the strengthening the reporting of observational studies in epidemiology (STROBE) statement [14] for observational studies, and the PRISMA checklist for systematic reviews and meta-analyses [15]. The NICE RCT checklist provides an assessment of potential bias in 4 categories: selection, performance, attrition and detection. The STROBE checklist contains 22 items that assess completeness of reporting in observational studies and the 27-item PRISMA checklist provides a similar assessment for systematic reviews and meta-analyses. The information collected in these checklists enabled a decision to be made about the eligibility of the studies for inclusion in this project. A second, independent reviewer performed a quality review check on a random sample of 20 % of the articles.

## Results

### Literature search

**Fig. 1:**
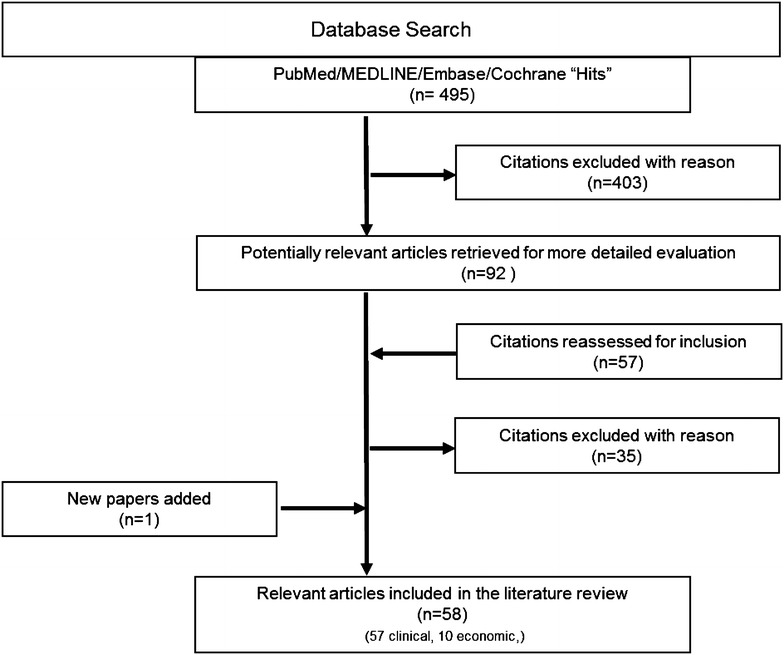
Clinical/economic burden literature search results

**Fig. 2:**
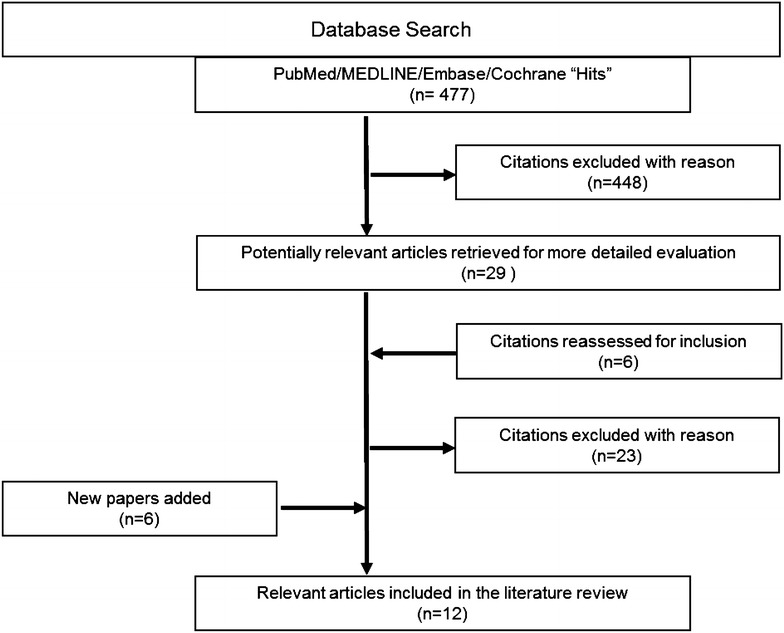
Humanistic literature search results

A total of 495 studies were identified by the clinical and economic burden literature searches with 58 studies being suitable for inclusion (Fig. 1). The 58 studies included: 3 systematic review/meta-analyses, 5 RCTs, and 27 cohort, 18 cross-sectional, and 5 case–control studies. A total of 477 studies were identified by the humanistic burden literature searches of which 12 studies were ultimately included (Fig. 2). The study designs of the 12 included articles were 6 RCTs, 4 cross-sectional and 2 case–control studies.

### Quality assessment

The clinical and economic burden literature included 3 systematic review/meta-analyses which met most of the PRISMA checklist criteria [16–18]. The criteria that were not met included: no description of methods for combining studies (100 %), not addressing risk of bias across studies (67 %) or individual studies (33 %) and not describing study limitations (67 %). Of the 5 RCTs appraised using the NICE RCT methodology checklist, most were rated as having a low risk of bias; however, a high risk of attrition bias was noted for three studies [19–21]. Lastly, the 50 remaining studies were assessed using the STROBE checklist. Many of the cohort studies did not indicate the study design (36 %), lacked reporting sensitivity or sub-group analyses (71 %), and missing or follow-up data was infrequently addressed (68 and 39 % respectively). The methodological limitations identified for the cross-sectional and case–control studies were very similar.

The humanistic burden literature included a total of 6 RCTs which were appraised by the NICE RCT methodology, all of which had an overall low risk of bias. The remaining 6 studies met most of the STROBE criteria; however, only 2 of the 6 studies adequately described the study setting [22, 23], 2 studies discussed efforts to address sources of bias [22, 24], and there was an overall lack of reporting on how missing data was addressed as well as sub-group and sensitivity analysis [23–27].

### Clinical burden evidence results

#### Overview

Of the 57 articles with clinical burden data (Tables 1, 2 and 3), the primary data source for 60 % of the studies (retrospective cohort and cross-sectional designs) was the provincial healthcare databases containing hospital records and pharmaceutical claims. The time frame of the included studies varied based on the study design. In general, the prospective designed studies included a much shorter time frame than systematic reviews or retrospective database analyses which often spanned decades.

**Table 1:** Summary of emergency department visit evidence

References	Geographic region (study years)	Patients	Arms or cohorts	Patients with ED visits, # (%)	# of ED visits	Mean annual ED # visits/patient
Polisena et al. [17]	Calgary (1998–2009)	≥60 years (mean age)	Telephone support	40.6 % of patients		0.1 visits
			Usual care	63.15 % of patients		0.4 visits
Labrecque et al. [29]	Montreal (2004)	40–75 years, stable COPD	Self-management education (n = 57)			Pre-index 1.1 visits Post-index 0.2 visits
			Usual care (n = 45)			Pre-index 0.4 visits Post-index 0.4 visits
Chapman et al. [1], Wouters et al. [37]	Canada (1 year study)	Diagnosed/undiagnosed COPD patients	All patients (n = 401)	70 patients (17.5 %)	151 visits	0.38 visits
Moullec et al. [28]	Montreal (2004–2006)	Montreal hospital patients	Integrated care (n = 96)	Prior year 27 (28.1 %), Post-Index 28 (29.2 %)		Pre and Post-Index 0.5 visits
			Usual care (n = 93)	Prior year 27 (29.0 %), Post-Index 26 (28.0 %)		Pre and Post-Index 0.5 visits
Bischoff et al. [39]		COPD patients, ≥40 years	All patients (n = 119)			Preceding year 0.65 visits
Rowe et al. [34]	US, Canada	≥55 year, stable COPD	Canadian patients (n = 63)			Previous year 1.0 visits
Tsai et al. [45]	US, Canada	≥55 year, stable COPD	Underweight (BMI <18.5, n = 50)			Previous year 2.0 visits
			Normal weight (BMI 18.5–24.9, n = 148)			Previous year 1.0 visits
			Overweight (BMI 25–29.9, n = 105)			Previous year 1.0 visits
			Obese (BMI ≥ 30, n = 92)			Previous year 1.0 visits
Sin et al. [38]	(1992–1997)	age ≥65 years, discharged diagnosis of COPD	No inhaled corticosteroid (n = 11,139)			Preceding year 1.2 visits
			Inhaled corticosteroid (n = 11,481)			Preceding year 1.6 visits
Johnston et al. [32]	Hamilton, Ontario (Dec 2006–Jan 2007)	≥40 year, COPD of mixed severity	GOLD stratum 0 (n = 39)			Previous year 1.6 visits
			GOLD stratum 1 and 2 (n = 31)			Previous year 1.4 visits
			GOLD stratum 3 and 4 (n = 44)			Previous year 1.8 visits
Wang et al. [35]	Montreal (2 year study)	≥40 years, Moderate-severe COPD hospitalized	All Patients (n = 282)	54 patients (19.1 %)	99 visits	1.82 visits
Rowe et al. [30], Rosychuk et al. [31]	Alberta (1999–2005)	≥55 years	All patients (38,638)	38,638 patients	85,330 visits	2.2 visits
Golmohammadi et al. [36]	Edmonton (2000–2002)	>45 years	Rehab program: DSS-S1 (n = 31)			Pre 42.1 visits/100 pt-years Post 13.6 visits/100 pt-years
			Rehab program: DSS-S2A (n = 78)			Pre 57.0 visits/100 pt-years Post 44.8 visits/100 pt-years
			Rehab program: DSS-S2B (n = 51)			Pre 29.5 visits/100 pt-years Post 16.3 visits/100 pt-years
			Rehab program: DSS-S3 (n = 41)			Pre 41.0 visits/100 pt-years Post 54.4 visits/100 pt-years
Stephenson et al. [40]	Ontario (2003–2010)	>66 years, Concomitant dementia and COPD	ChEI users (n = 7166)	Baseline 538 (7.5 %)		
			ChEI non-users (n = 7166)	Baseline 517 (7.2 %)		
Blais et al. [41]	Quebec (Feb 2003–Jan 2007)	≥40 years	Budesonide/formoterol (n = 1131)	10.3 % of patients	182 visits	
			Propionate/salmeterol (n = 1131)	13.1 % of patients	256 visits	
Gershon et al. [42]	Ontario (2003–2007)	≥66 years	Long-acting anticholinergic (n = 28,563)	12.2 % of patients		
			Long-acting beta-agonist (n = 17,840)	11.7 % of patients		
FitzGerald et al. [43]	Canada (1 year study)	≥40 year	All patients (n = 609), all exacerbation (n = 691)		193 visits	
			Patients with exacerbations (n = 278)	111 patients (39.9 %)		
Sedeno et al. [21]		COPD patients	Usual care (n = 81)	54.4 % patients		
			Self-management group (n = 85)	29.9 % patients		
Bourbeau et al. [44]		Advanced COPD, ≥1 hospitalization for exacerbation in last year	Usual care (n = 95)	63.2 % patients		
			Self-management care (n = 96)	40.6 % patients		
Mittmann et al. [3]	Canada	Moderate and severe COPD exacerbations	All patients (n = 609), all exacerbation (790 exacerbations)		245 visits	
			Moderate exacerbation (639 exacerbations)		105 visits	
			Severe exacerbation (151 exacerbations)		140 visits	
Beauchesne et al. [77]	(1995–2004)	COPD patients	Home management program (n = 152)		29 visits	
Dormuth et al. [60]	British Columbia	≥45 years, 2.5-year period after public coverage	Predicted use		6658 visits	
			Observed use		7434 visits

**Table 2:** Summary of hospitalization evidence

References	Geographic region (study years)	Patients	Arms or cohorts	Hospitalization, # or % patients	# of hospital visits	Mean annual hospital # visits/patient
Tsai et al. [45]	US, Canada	≥55 year, stable COPD	Underweight (BMI <18.5, n = 50)			Prior year 0 visits
			Normal weight (BMI 18.5–24.9, n = 148)			Prior year 0 visits
			Overweight (BMI 25–29.9, n = 105)			Prior year 0 visits
			Obese (BMI ≥ 30, n = 92)			Prior year 0 visits
Rowe et al. [34]	US, Canada	≥55 year, stable COPD	Canadian patients (n = 63)			Prior year 0 visits
Johnston et al. [32]	Hamilton, Ontario (Dec 2006–Jan 2007)	≥40 year, COPD of mixed severity	GOLD stratum 0 (n = 39)		1	Prior year 1.3 visits
			GOLD stratum 1 and 2 (n = 31)		3	Prior year 1.2 visits
			GOLD stratum 3 and 4 (n = 44)		7	Prior year 1.5 visits
Labrecque et al. [29]	Montreal (2004)	40–75 years, stable COPD	Self-management (n = 57)			Prior year 0.7 visits Post index 0.3 visits
			Usual care (n = 45)			Prior year 0.5 visits Post index 0.5 visits
Moullec et al. [28]	Montreal (2004–2006)	Montreal hospital patients	Integrated care (n = 96)	Prior year 96 Post-index 38		Prior year 1.3 visits Post index 0.7 visits
			Usual care (n = 93)	Prior year 69 Post-index 55		Prior year 1.5 visits Post-index 1.3 visits
Ohinmaa et al. [46]	Alberta	Adult from Canadian Community Health Survey	20–44 years			0 visits
			45–64 years			3.45 visits
			>65 years			5.19 visits
Blais et al. [41]	Quebec (Feb 2003–Jan 2007)	≥40 years	Budesonide/formoterol (n = 1131)	8.6 %	130	0.11 visits
			Propionate/salmeterol (n = 1131)	12.4 %	233	0.21 visits
FitzGerald et al. [43]	Canada (1 year study)	≥40 year,	All patients (n = 609)	75	112	0.2 visits
			Patients with exacerbations (n = 278)		75	1.5 visits
Chapman et al. [1], Wouters et al. [37]	Canada (1 year study)	Diagnosed/undiagnosed COPD patients	All patients (n = 401)	Prior year 14 %		0.32 visits (0 visits/year 1999–2005)
Wong et al. [47]	Vancouver, British Columbia (winter 2006–2007)	Admitted St. Paul’s Hospital with AECOPD diagnosis	Entire population (n = 109)			3.3 visits (6-month readmission rate)
Beaulieu et al. [48]		Moderate-severe COPD	Self-administered prescription (n = 46)			0.3 visits (prior 6-months)
			Control (n = 43)			0.5 visits (prior 6-months)
Sin et al. [38]	(1992–1997)	age ≥65 years, discharged diagnosis of COPD	All Patients (n = 22,620)	5654 (25 % repeat hospitalization)		
Chen et al. [50]	(First admission 1999–2000)	COPD In-patients, ≥40 years	Entire population (n = 108,726)	49.1 % rehospitalization		
Huiart et al. [51]	(1990–1997, 1st COPD treatment)	≥55 years, first treatment of COPD	All (n = 5648)	1027	2326	101.4 visits/1000 PY
			Female (n = 2606)	399	812	74.3 visits/1000 PY
			Male (n = 3042)	673	1514	126.1 visits/1000 PY
Sedeno et al. [21]		COPD patients	Usual care (n = 81)	36.3 %		
			Self-management group (n = 85)	17.2 %		
Chen et al. [76]		General population, broad (B) and narrow (N) defined cases for COPD hospitalization	All (n = 6,099,756)	B = 257,604, N = 85,189		B = 42.2, N = 14.0/1000 PY
			Age 55–59 (n = 1,332,254)	B = 16,671, N = 5129		B = 12.5, N = 3.8/1000 PY
			Age 60–64 (n = 1,207,873)	B = 26,904, N = 8579		B = 22.3, N = 7.1/1000 PY
			Age 65–69 (n = 1,121,508)	B = 40,823, N = 13,404		B = 36.4, N = 12.0/1000 PY
			Age 70–74 (n = 963,007)	B = 51,782, N = 17,310		B = 53.8, N = 18.0/1000 PY
			Age 75–79 (n = 683,520)	B = 49,788, N = 16,983		B = 72.8, N = 24.8/1000 PY
			Age 80–84 (n = 450,458)	B = 40,666, N = 13,844		B = 90.3, N = 30.7/1000 PY
			Age 85–89 (n = 227,533)	B = 21,676, N = 7,046		B = 95.3, N = 31.0/1000 PY
			Age 90+ (n = 113,603)	B = 9294, N = 2894		B = 81.8, N = 25.5/1000 PY
Tu et al. [78]		Active smoking adults, ≥15 years of age				167 visits (predicted, linear regression)
Curkendall et al. [79]	(1997–2000)	≥40 years, COPD diagnosed with ≥2 bronchodilators within 6-months	COPD (n = 11,493)			598.36/1000 PY CV related; 109.5/1000 PY
			Controls (n = 22,986)			221.23/1000 PY CV related; 44.66/1,000 PY
Mittmann et al. [3]	Canada	Moderate and severe COPD exacerbations	All exacerbations (n = 609)		151	
			Moderate exacerbation		140	
			Severe exacerbation		151	
Mancini et al. [53]		COPD patients	Coronary revascularization (n = 946)	Prior year 2.6–5.9 %		
			Without MI (n = 18,774)	Prior year 1.6–7.3 %		
Gonzalez et al. [80]		>66 years, received ≥3 respiratory medications	Women (n = 19,260)	Prior year 2.7 %		
			Men (n = 23,893)	Prior year 2.6 %		
Macie et al. [81]	Manitoba (1997–2000)	Drug claim for obstructive airways disease	All recipients (n = 6,041)	3.2 %		
			Control (n = 60,410)	5.2 %		
Ernst et al. [82]		Hospitalized with Pneumonia	Case (n = 23,942)	14.5 %		
			Control (n = 95,768)	3.6 %		
Chan et al. [20]		COPD Diagnosis	Tiotropium (n = 608)	8.4 %		
			Placebo (n = 305)	8.2 %		
Gershon et al. [42]	Ontario (2003–2007)	≥66 years	Long-acting anticholinergic (n = 28,563)	33.3 %		
			Long-acting beta-agonist (n = 17,840)	30.7 %		
Monfared et al. [83]	(1990–1996)	Elderly COPD patients	RAMQ database (n = 1233)	32.7 %		
			MED-ECHO database (n = 1206)	32.0 %		
Polisena et al. [17]	Calgary (1998–2009)	≥60 years (mean age)	Telephone support	32–46 %		
			Usual Care	51–66 %		
Goodridge et al. [52]	(Deceased in 2004)	COPD or lung cancer death	All patients (n = 1098)	80.4 %		
Aaron et al. [56]	Canada (1995–2004)	COPD patients	Tiotropium + Plac (n = 156)	62		
			Tiotropium + Salmeterol (n = 148)	48		
			Tiotropium + Fluticasone-Salmeterol (n = 145)	41		
Benayoun et al. [84]	(1996–1997)	>45 years, initiating treatment with combination inhaler	Combined Bronchodilator (n = 641)	Prior year 202		
			Double-users (n = 411)	Prior year 279		
Stephenson et al. [40]	Ontario (2003–2010)	>66 years, Concomitant dementia and COPD	ChEI users (n = 7166)	Prior year 469		
			ChEI non-users (n = 7166)	Prior year 403		
Bourbeau et al. [85]		≥55 years, without asthma initiating COPD treatment	Case (n = 843)	Current ICS use 275, past user 141		
			Control (n = 11,030)	Current ICS use 2994, past user 1357		
Beauchesne et al. [77]	(1995–2004)	COPD patients	Home management (n = 152)		100	
Bourbeau et al. [44]		Advanced COPD, ≥1 hospitalization for exacerbation in last year	Usual care (n = 95)		Prior, 152 Year 1, 118	
			Self-management care (n = 96)		Prior year, 158 Year 1, 71	
Disano et al. [86]	(2003–2006)	Ambulatory care COPD	Low SES		381^a^	
			Average SES		210^a^	
			High SES		129^a^	
Keenan et al. [87]	London	COPD with exacerbation at emergency room	All patients (n = 25)		355 (over 3 years 2 months)	
Dormuth et al. [60]	British Columbia	≥45 years, 2.5-year period after public coverage	Predicted use		42,735	
			Observed use		44,007	

*PY* patient years

^a^Rates per 100,000 people

**Table 3:** Summary of physician visit evidence

References	Geographic region (study years)	Patients	Arms or cohorts	Physician visits, # or % patients	# of physician visits	Mean annual physician visits, # visits/patient
Blais et al. [41]	Quebec (Feb 2003–Jan 2007)	≥40 years of age	Budesonide/formoterol (n = 1131)	58.5 %	1956	1.73 visits
			Propionate/salmeterol (n = 1131)	59.7 %	1779	1.57 visits
Ohinmaa et al. [46]	Alberta	Adult from Canadian Community Health Survey	20–44 years			6.52 visits
			45–64 years			5.63 visits
			>65 years			8.10 visits
Goodridge et al. [52]	(Deceased in 2004)	COPD or lung cancer death	All patients (n = 1098)	59.8 % (>24 visits within 12 months of death)		28.0 visits (12 months prior to death)
Polisena et al. [17]	Calgary (1998–2009)	≥60 years of age	Telephone support			PCP; 0.48 vs. 1.18 UC Office visits; 5.0 vs. 6.0 UC
			Home telemonitoring			Office visits; 3.2 vs 2.3 UC
Rowe et al. [34]	US, Canada	≥55 year, stable COPD	Canadian patients (n = 63)			0 urgent clinic visits, prior-year
			US patients (n = 334)			0 urgent clinic visits, prior-year
Sin et al. [38]	(1992–1997)	age ≥65 years, discharged diagnosis of COPD	No-inhaled corticosteroid (n = 11,139)			4.1 visits, prior year
			Inhaled corticosteroid (n = 11,481)			4.1 visits, prior year
Mancini et al. [53]		COPD patients, with CV revascularization and without MI newly treated with NSAIDS	High-risk cohort cases (n = 946)			20 visits, prior year
			High-risk controls (n = 18,774)			19 visits, prior year
			Low-risk cohort cases (n = 4907)			5 visits, prior year
			Low-risk controls (n = 98,097)			5 visits, prior year
Beaulieu et al. [48]		Moderate-severe COPD	Self-administered Rx (n = 46)			0.8 visits (prior 6-months)
			Control (n = 43)			0.7 visits (prior 6-months)
Johnston et al. [32]	Hamilton, Ontario (Dec 2006–Jan 2007)	≥40 year, COPD of mixed severity	GOLD stratum 0 (n = 39)		9	
			GOLD stratum 1 and 2 (n = 31)		15	
			GOLD stratum 3 and 4 (n = 44)		15	
Bourbeau et al. [44]		Advanced COPD, ≥1 hospitalization for exacerbation in last year	Usual Care (n = 95)		Scheduled 309 Unscheduled 112	
			Self-management care (n = 96)		Scheduled 354 Unscheduled 46	
Sedeno et al. [21]		COPD patients	Usual care (n = 81)	30.9 %		
			Self-management group (n = 85)	8.2 %		
Bischoff et al. [39]		COPD patients, ≥40 years	All patients (n = 217)	Unscheduled Visits; 70		
Chapman et al. [1], Wouters et al. [37]	Canada, 7 countries North America and Europe (1 year study)	Diagnosed/undiagnosed COPD patients	All patients (n = 401)	Scheduled PCP; 225 Unscheduled PCP; 175	Scheduled 1506 Unscheduled 175	
Macie et al. [81]	Manitoba (1997–2000)	Drug claim for obstructive airways disease	All recipients (n = 6041)	0–1 visit; 18.0 2–3 visits; 23.0 % 4–9 visits; 36.6 % ≥10 visits; 22.4 %		
			Control (n = 60,410)	0–1 visit; 32.8 % 2–3 visits; 24.0 % 4–9 visits; 29.1 % ≥10 visits; 14.1 %		
Disano et al. [86]	Canada (fiscal years 2003–04, 2004–05 and 2005–06)	Children under 20 years, fiscal years 2003–04, 2004–05 and 2005–06	All (46,173)	48 %		
			Underweight (BMI <18.5)	42 %		
			Normal weight (BMI 18.5–24.9)	56 %		
			Overweight (BMI 25–29.9)	55 %		
			Obese (BMI ≥ 30)	32 %		
FitzGerald et al. [43]	Canada (1 year study)	≥40 years of age	Patients with exacerbations (n = 278)	255		
Stephenson et al. [40]	Ontario (2003–2010)	>66 years, Concomitant dementia and COPD	ChEI users (n = 7166)	1 visit, 36; ≥2 visits 7062		
			ChEI non-users (n = 7166)	1 visit, 131; ≥2 visits 6940		
Dormuth et al. [88]	British Columbia (1997–2004)	≥65 years of age	Policy Group (n = 19,985)	6-months prior/follow-up 0–4 visits; 4610 ≥5 visits; 15,375		
			Pre-policy group (n = 17,335)	6-months prior/follow-up 0–4 visits; 4439 ≥5 visits; 12,896		
Mittmann et al. [3]	Canada	Moderate and severe COPD exacerbations	All exacerbations (n = 609)		618	
			Moderate exacerbation		574	
			Severe exacerbation		44	
Sin et al. [89]	Alberta (1996–1997)	General Population (2.8 million)	Aboriginals		15,712	
			Non-aboriginals		275,134	
Dormuth et al. [60]	British Columbia	≥45 years, 2.5-year period after public coverage	Predicted use		2,073,233 (over 2.5 years)	
			Observed use		2,094,360 (over 2.5 years)	
Rowe et al. [30]	Alberta (1999–2005)	≥55 years at time of ED visit	All Patients (n = 7302)		GP 107,405 Int Med 13,907 Resp Med 5287	
Moineddin et al. [90]	Ontario (1992–2002)	All patients with at least 1 primary care visit			4,662,735 over 11 years	

*PY* patient years, *PCP* primary care physician, *UC* usual care, *Rx* prescription

#### Emergency department (ED) visits

Emergency department visits were reported as an outcome in 23 out of the 58 studies (Table 1). A number of studies reported the mean number of emergency department visits which ranged from 0.1 to 2.20 per year [1, 17, 28–39]. Eleven studies reported that 7.2–63.2 % of patients with COPD visited the emergency department [1, 17, 21, 28, 30, 35, 40–44]. Johnston [32] reported the mean annual number of ED visits by disease severity. The instrument used to assess disease severity was developed by the global initiative for chronic obstructive lung disease (GOLD) and categorizes patients from mild to very severe in 4 levels (GOLD 1–4 stratum). The mean number of annual ED visits ranged from 1.4 (GOLD stratum 1 and 2) to 1.8 (GOLD stratum 3 and 4) in COPD patients with an exacerbation [32].

Three studies reported how different pre/post interventions affected ED visits in COPD patients. Overall ED visits were less in COPD patients with self-management education or self-care management programs; however, integrated care appeared to provide no benefit on the annual mean number of ED visits [28, 29, 44].

#### Hospitalization

Hospitalization was reported as an outcome in 38 of the 58 studies (Table 2). The rates were reported as either pre- or post- index hospitalizations. The mean number of annual hospital visits per COPD patient per year ranged from: 0–1.5 pre-index to 0–5.19 post-index [1, 28, 29, 32, 34, 41, 43, 45–48]. Three studies reported the rates of hospitalization according to disease severity and/or COPD exacerbations and found higher rates of hospitalization in more severe patients (GOLD stratum 3 or 4) and those with more severe exacerbations [3, 32, 43]. Hospital readmission rates varied between three studies with Sin [49] reporting a rate of 25 % for COPD patients ≥65 years of age, Chen [50] reporting a rate of 49.1 % in patients ≥40 years of age, and Wong [47] reporting 3.3 mean annual number of hospital readmissions in patients with a diagnosis of AECOPD.

The relationship of COPD hospitalization rates to patient demographic characteristics was examined in three studies. A higher rate of hospitalization was found in male COPD patients [126.1/1000 patient years (PY)] than females (74.3/1000 PY) and in those >65 years of age (5.19 visits/patient annually) versus those 45–64 years of age (3.45 visits/patient annually) [46, 51]. One study found that COPD patients’ body mass index (BMI) status had no effect on hospitalization rates [45].

Lastly, three studies examined the effects of different interventions on hospitalization rates in COPD patients. Moullec [28] found that integrated care (a combination of self-management education and case management) resulted in a decreased mean number of hospitalizations compared to usual care. Lebrecque [29] and Sedeno [21] found that self-management interventions also reduced hospitalizations compared to usual care.

#### Physician visits

A total of 24 studies reported the rate of physician visits for COPD (Table 3). The annual rate of physician visits post-index for COPD patients ranged between 1.57 and 28 visits annually [41, 46, 52]. Two studies found that elderly COPD patients (>65 years) had high rates of physician visits compared to younger patients (from 4.1 to 8.1 visits/year) [38, 46], one study found those at high risk for CV-related comorbidities had higher physician visit rates compared to those with low risk (20 vs. 5 visits per year) [53], and one study reported that COPD patients diagnosed with GOLD stratum 1–4 had a higher number of exacerbations requiring a physician visit compared to those with GOLD stratum 0 (15 vs. 9 visits, respectively) [32]. Goodridge [52] found the highest rate of physician visits for COPD patients was within 12 months of death (28 visits/year) and Rowe [34] found that Canadian and US stable COPD patients had similar mean annual urgent clinic visit rates. Lastly, two studies found that self-management interventions reduced the number of unscheduled physician visits [21, 44] and a review article found a reduction in the number of annual physician visits for patients receiving telephone support [17].

### Humanistic burden evidence

#### Overview

A total of 12 studies were identified describing the humanistic burden by measuring the effect of COPD on a patient’s health-related quality of life (HRQoL) and physical activity (Table 4). Study timeframes were not reported in three studies and variation was found in the definition of COPD across all studies. With regard to the type of HRQoL instruments used, 4 studies [22, 25, 54, 55] reported outcomes for the 36-item short form health survey (SF-36) and 5 studies reported results for The St. George Respiratory Questionnaire (SGRQ) [20, 22, 27, 54, 56]. Other scales that were used to assess HRQoL were the chronic respiratory disease (CRD) Index Questionnaire, the sickness impact profile (SIP) and the Chronic Respiratory Questionnaire (CRQ).

**Table 4:** Summary of humanistic burden evidence

References/study period	Patient group	N	Scale	Baseline score, mean (SD)	Endpoint score, mean (SD)	Change from baseline, mean (SD)
SF-36 score						
Appleton et al. [54]/November 1999–September 2001	Salmeterol	172	PH	36.5 (10.0) [N = 146]	37.1 (10.5) [N = 131]	0.3 (7.7) [N = 146]; mean difference vs placebo: 0.30; 95 % CI (−1.3, 1.9)
			ME	49.3 (10.8) [N = 146]	50.0 (10.5) [N = 131]	1.1 (10.0) [N = 146]; mean difference vs placebo 0.03; 95 % CI (−1.9, 1.9)
	Placebo	176	PH	36.1 (9.5) [N = 156]	36.8 (10.3) [N = 144]	0.1 (6.4) [N = 156]
			ME	48.8 (11.0) [N = 156]	50.3 (10.6) [N = 144]	1.1 (9.1) [N = 156]
HajGhanbari et al. [25]/study period not reported	COPD patients	47	PH		35.2 (1.7) p = 0.000; AMD vs control: 16.9	
			ME		42.0 (1.8) p = 0.000; AMD vs control: 12.8	
	Healthy controls	47	PH		52.0 (1.3)	
			ME		54.7 (1.30)	
Moullec et al. [22]/Apr 2004–May 2006	Usual care	50	PH	37 (10)		
			ME	47 (12)		
	Intervention	60	PH	35 (8) p = 0.33		
			ME	45 (12) p = 0.26		
Lacasse et al. [55]/12 weeks	Paroxetine	12	PH	18.6 (10.0)		
			MH	53.1(23.2)		
	Placebo	11	PH	19.0 (9.9); p = 0.9		
			MH	58.0 (16.8); p = 0.4		
SGRQ scores						
Appleton et al. [54]/November 1999–September 2001	Salmeterol	172	T	46.2 (18.0) [N = 150]	41.6 (19.0) [N = 124]	−2.9 (11.1) [N = 150]
			S	59.6 (18.4) [N = 150	55.4 (19.7) [N = 124]	−3.0 (15.8) [N = 150]
			A	61.6 (21.9) [N = 150	53.3 (23.8) [N = 124]	−5.9 (15.4) [N = 150]
			I	33.5 (20.7) [N = 150]	30.6 (20.0) [N = 124]	−1.2 (13.6) [N = 150]
	Placebo	176	T	46.8 (16.6) [N = 157]	44.7 (18.6) [N = 139]	−1.3 (10.3) [N = 157]
			S	56.7 (19.6) [N = 157]	57.3 (21.6) [N = 139]	1.4 (15.5) [N = 157]
			A	62.7 (18.9) [N = 157]	59.7 (22.4) [N = 139]	−3.0 (15.0) [N = 157]
			I	34.8 (18.9) [N = 157]	32.4 (20.3) [N = 139]	−1.2 (11.5) [N = 157]
Aaron et al. [56]/October 2003–January 2006	Tiotropium + placebo	156	T			−4.5
			S			
			A			
			I			
	Tiotropium + salmeterol	148	T			−6.3, p = 0.02
			S			
			A			
			I			
	Tiotropium + fluticasone/salmeterol	145	T			−8.6, p = 0.01
			S			
			A			
			I			
Chan et al. [20]/1 year	Tiotropium	608	T		40.9	
			S		44.4	
			A			
			I		28.5	
	Placebo	305	T		43.7, p < 0.01	
			S		49.3, p < 0.01	
			A			
			I		31.3, p < 0.01	
Low et al. [27]/study period not reported	Patient	67	T		–	
			S		57.68 (24.71) [N = 66]; mean difference vs spouse: 1.73; p = 0.497	
			A		70.42 (17.44) [N = 67]; mean difference vs spouse: −0.21; p = 0.771	
			I		41.05 (22.83) [N = 66]; mean difference vs spouse: 5.6; p = 0.002	
	Spouse	67	T		–	
			S		59.41 (23.05) [N = 65]	
			A		70.21 (18.72) [N = 66]	
			I		47.29 (23.12) [N = 65]	
Moullec et al. [22]/Apr 2004–May 2006	Usual care	50	T	49 (18)		
			S	55 (16)		
			A	66 (23)		
			I	38 (20)		
	Intervention	60	T	48 (16), p = 0.72		
			S	54 (18), p = 0.74		
			A	65 (20), p = 0.85		
			I	37 (19), p = 0.72		
Chronic Respiratory Questionnaire Scores (CRQ)						
Lacasse et al. [55]/12 weeks	Paroxetine	12	TG			
			D	3.4 (0.9), p = 1.0		
			E	3.5 (0.9), p = 0.8		
			M	4.3 (1.0), p = 0.2		
			F	3.6 (0.8), p = 0.3		
	Placebo	11	TG	–		
			D	3.4 (0.6)		
			E	3.7 (1.0)		
			M	4.9 (0.9)		
			F	3.2 (1.1)		
Bourbeau et al. [57] 6 months	Budesonide	39	TG	–		
			D	19.9 (6.2)		−1.8 (−3.9 to 0.2)
			E	37.9 (6.9)		−1.9 (−5.3 to 1.4)
			M	21.4 (4.2)		−0.5 (−2.4 to 1.4)
			F	20.7 (3.6)		−3.0 (−4.9 to −1.2)
	Placebo	40	TG	–		–
			D	19.5 (5.8)		−0.5 (−2.3 to −1.3)
			E	36.2 (9.6)		−0.6 (−3.4 to 2.2)
			M	21.7 (5.8)		−1.3 (−3.0 to 0.5)
			F	19.3 (5.6)		−1.4 (−3.1 to 0.3)
Leigh et al. [24]/4-week treatment period	Overall population	40	TG	17.5 (3.6)	Post-PB: 18.1 (3.5) Post-BDN: 19.4 (3.4) Post-PDN: 21.0 (3.4)	
			D	3.7 (1.0)	Post-PB: 3.9 (0.9) Post-BDN: 4.4 (1.1) Post-PDN: 4.6 (1.3)	
			E			
			M			
			F			
Physical activity						
Vozoris et al. [23]/1994–2007	Obese COPD patients	858	Inactivity^a^; restricted activity^a^	Inactive: 68 % patients Activity restriction: 72 % patients		
	Non-obese COPD patients	2611	Inactivity^a^; restricted activity^a^	Inactive: 60 % patients Activity restriction: 60 % patients		
Rocker et al. [26]. Study period not reported	Severe, stable COPD patients	8	Palliative performance scale	Scores ranged from 50 to 70 %		

*A* activity score, *AMD* absolute mean difference, *BDN* budesonide, *D* dyspnea, *E* emotional function, *F* fatigue, *M* mastery, *ME* Mental Health summary score, *PB* placebo, *PDN* prednisone, *PH* Physical Health summary score, *S* symptoms score, *T* total score, *TG* total (Global) score, *I* impact score

^a^Canadian Fitness and Lifestyle Research Institute defined Inactivity as total daily energy expenditure value of < 1.5 kcal/kg/day; restricted activity: sometimes or often had difficulty with simple activities such as walking, climbing stairs, and bending

#### Sf-36

Of the 4 studies reporting SF-36 evidence, one study found that COPD patients receiving salmeterol did not experience significant improvement in their SF-36 mental or physical health summary scores compared to baseline [54]. In contrast, a case–control study reported an absolute mean difference of 16.9 in the SF-36 physical health summary score and 12.8 in the mental component score for COPD patients compared to healthy controls. The study also indicated a significantly worse (p < 0.001) level of functioning for patients with COPD [25].

#### St George’s Respiratory Questionnaire (SGRQ)

Four of the five studies reporting SGRQ data compared an intervention to placebo or usual care in a COPD population [20, 22, 54, 56], while one study reported data for COPD patients versus their spouses [27]. Three RCTs found pharmaceutical agents (tiotropium, salmeterol, tiotropium plus salmeterol and tiotropium plus fluticasone/salmeterol) significantly improved patients’ quality of life as measured by the SGRQ score [20, 54, 56]. Of the remaining two studies, one cross-sectional survey found a significant mean difference (5.6, p = 0.002) for the SGRQ impact of disease scores between COPD patients and their non-COPD spouse [27] and a prospective, observational study reported no significant differences in SGRQ scores at baseline between the self-management education program and usual care groups [22].

#### Chronic Respiratory Questionnaire (CRQ)

Three studies used the CRQ to assess the quality of life of COPD patients utilizing different pharmaceutical interventions (paroxetine, budesonide, prednisone). Of the three studies, paroxetine (CRQ emotional function domain) and inhaled corticosteroids (budesonide) were found to produce significant improvements in patients’ quality of life; however, inhaled corticosteroids (even in ‘high’ doses) did not appear to provide significant HRQoL improvement over that achieved with oral prednisone [24, 55, 57].

#### Miscellanous HRQoL instruments

Several studies utilized additional HRQoL instruments to assess the quality of life of COPD patients. A study by HajGhanbari [25] found that pain severity [measured by the McGill Pain Questionnaire (MPQ) and brief pain inventory scale (BPI)] showed moderate to strong negative correlations to the physical component score of the SF-36 (−0.45, −0.61, −0.70, respectively; p < 0.001). In addition, a cross-sectional survey study using the SIP found significant differences in the mean score between patients’ and healthy spouses’ ratings of the SIP physical score (p = 0.009), but non-significant differences in psychosocial score (p = 0.497) [27]. Finally, a single RCT conducted by Aaron [58] using the chronic respiratory disease index HRQoL instrument (CRD) found that prednisone use did not result in a significant (p = 0.14) overall health benefit (total score) when compared to placebo, although prednisone reduced the incidence of relapse and improved both lung function and dyspnea.

#### Physical activity

Three studies reported on physical activity related to the burden of COPD. A cross-sectional study using the Canadian national health survey data (1994–2007) found that approximately 68 % of obese and 60 % of non-obese COPD patients were inactive. Additionally, approximately 72 % of obese and 60 % of non-obese COPD patients reported activity restriction [23]. Furthermore, a cross-sectional study by Rocker [26] in patients with severe, stable COPD found that scores on the palliative performance scale from semi-structured interviews ranged from 50 to 70 % and that all patients had a score of 5 on the Medical Research Council dyspnea scale (i.e., they were too short of breath to leave their homes or were breathless when dressing or undressing). The significance of pain in COPD patients was reflected in pain-related interference in activities, which may partly account for the lower SF-36 physical component scores in HRQoL and the lower physical activity scores on the community health activities model program for seniors (CHAMPS) questionnaire [25].

### Economic burden evidence

#### Overview

A total of 5 studies contained outcomes of interest and were included in this review. Of the 5 studies, 4 studies reported the patient level direct costs and 2 studies reported population level direct costs for COPD patients (Tables 5, 6).

**Table 5:** Summary of average annual patient level direct costs evidence (CAN$)

References (study period)	Categories	Patient group	Patient perspective inflated cost/patient	Societal perspective inflated cost/patient
Chapman et al. [1] (12 months)	All	All	$2444.17	$3910.39
	Gender	Male	$1941.09	$2817.88
		Female	$2926.30	$4956.03
	Smoking status	Former smokers	$3348.67	$4702.55
		Current smokers	$1357.06	$2958.41
	Comorbidities	Yes	$2506.92	$4568.22
		No	$2370.68	$3127.96
	Education status	Less educated	$3043.48	$4540.89
		More educated	$2142.85	$3638.46
Wouters et al. [37] (1 year)	All	All	$2378.59	$6693.37
	Gender	Male	NR	$2741.62
		Female	NR	$4254.24
	Smoking status	Former smokers	NR	$4575.67
		Current smokers	NR	$2877.75
	Education status	Less educated	NR	$4418.73
		Well educated	NR	$3539.53
Mittmann et al. [3] (52 weeks)	Moderate exacerbation	ITT population	$718.48	NR
		Clinically evaluable population	$847.38	NR
	Severe exacerbation	ITT population	10,712.14	NR
		Clinically evaluable population	11,156.01	NR
Maleki-Yazdi et al. [59] (Oct 2009 and Jan 2010)	All	All	$4391.16	NR
	Acute exacerbation	Clinically evaluable population	$3214.75	NR

*ITT* intention to treat, *NR* not reported

**Table 6:** Summary of average annual population level direct costs evidence (CAN$)

References (study period)	Population	Resource	Inflated 2012 CAN$
Dormuth et al. [60] (Jul 2007–Dec 2009)	Residents of British Columbia, 45+ years old	Medication (inhaled anticholinergic)	$26,298,835.28 (ministry paid: $13,276,279.45, out of pocket: $13,022,555.82)
		Any hospital admission	$310,494,472.10
		Emergency COPD admission	$59,456,281.50
Mittmann et al. [3] (52 weeks)	Mean age of 68.6 years	Moderate exacerbation	$182.7–$254.44 million
		Severe exacerbation	$469.64–$642.26 million

#### Patient level direct costs

Overall, the average total cost per patient was reported from both a patient perspective and a society perspective (accounting for inflation) and ranged between CAN $2444.17–CAN $4391.16 (patient perspective) and CAN $3910.39–CAN $6693.37 (societal perspective) annually. The average cost per acute COPD exacerbation reported by Mittmann [3] and Maleki-Yazdi [59] ranged from $718–$11,156 and the cost was found to increase with the severity of the exacerbation. No studies were found to examine the relationship of cost to overall disease severity.

Two studies examined differences in costs based on patient characteristics. Chapman [1] and Wouters [37] both reported female COPD patients incurred more costs compared to male patients from both a patient and a societal perspective (additional $985/patient from a patient perspective, $1513–2138/patient from a societal perspective). In addition, these studies also found that former smokers incurred more costs than current smokers (additional $1992/patient from a patient perspective, $1698–$1744/patient from a societal perspective) and that COPD patients with less education incurred more costs than those who are more highly educated (additional $901/patient from a patient perspective, $879–902/patient from a societal perspective). Lastly, Chapman [1] reported that patients with comorbidities were more costly than those without comorbidities (additional $136/patient from a patient perspective, $1440/patient from a societal perspective).

#### Population level direct costs

Population level direct costs (in Canadian dollars) were examined in two studies (Table 6). Dormuth [60] found that residents of British Columbia who were dispensed an inhaled anti-cholinergic (IAC) medication (ipratropium or tiotropium) cost $26,298,835 annually over 2.5 years for IACs (Ministry of Health $13,276,279, out of pocket $13,022,556), $310,494,472 for any hospital admission and $59,456,281 for emergency COPD admissions over the 2.5 year period. The second study by Mittmann [3] estimated that moderate COPD exacerbations cost $182.70–$254.44 million annually while severe exacerbations cost $469.64–$642.26 million annually in Canada.

## Discussion

COPD is one of the world’s most common health problems [2]. This review found evidence that the clinical, economic and humanistic burden of COPD is substantial in Canada. COPD patients were found to average 0–4 annual emergency department visits, 0.3–1.5 annual hospital visits, and 0.7–5 annual physician visits which are similar to the rates reported worldwide. Variance in these rates across studies may reflect population differences, methodological differences and/or treatment pattern differences between studies. In Canada, the health care services are provided by the private sectors but they are delivered through publicly funded health care systems. For instance, basic services such as physician care are provided by private doctors but the physician fees are paid for by the government. Hospital care is delivered by publicly funded hospitals which are mostly independent institutions incorporated under provincial Corporations Acts. The universal health care system, however, does not include coverage of prescription medication; drug benefit plans for eligible groups are available under provincial and territorial governments.

In terms of ED services, an international survey found that around the world, the percentage of COPD patients using ED services ranges from 1 % (China) to 25 % (Brazil) [61]. The relatively small number of ED visits found for Canadian COPD patients would suggest that the use of ED services for COPD patients may fall on the lower end worldwide. Hospitalization rates, hospital readmission rates, and the number of physician visits for Canadian COPD patients were found to be consistent with rates found in the US [62–64]. Additionally, trends of increasing healthcare resource use as COPD worsens are consistent with worldwide data [61, 65].

Primary care has been reported to have the greatest proportion of worldwide burden in the treatment of COPD. Furthermore, increasing severity of COPD imposes a greater burden on the use of primary care resources [61]. Evidence was found that self-care management programs may help with reducing the number of ED visits, hospitalizations, and physician visits. Additionally, telephone support services were found to reduce the number of physician office visits. Integrated care programs, however, appear to reduce the mean number of hospitalizations but not ED visits.

COPD has a profound impact on patients’ quality of life [66]. Evidence found in this review, while not overwhelming, found that Canadians with COPD have a poorer quality of life. Worldwide data suggests that up to 45 % of COPD patients experience pain and that increases in pain are associated with disease progression [67–72]. The significance of pain in COPD patients was reflected in greater pain-related interference on activities of daily living. In the Canadian Hidden Depths survey, COPD symptoms were found to have a significant effect on a range of daily activities (including climbing stairs, housework, getting dressed and sleeping) for a majority of respondents [73]. Clinicians face challenges in treating COPD related pain in that opioids, common pharmacotherapy, are not recommended for use in COPD patients, presumably due to their effects on the reduction of breathing rates which may further exacerbate COPD [4]. Additionally, this review found evidence that 60–72 % of COPD patients are inactive and/or have activity restrictions with obese patients having the highest percentages.

Obesity is one of the leading causes of overall morbidity and mortality [74, 75]. Thus it is not surprising that health consequences of obesity are seen in the COPD population and coupled with progressively worsening lung function. It is therefore important that more research is performed in order to better understand the impact of interventions on the quality of life and how to maximize patient functioning.

Data from this review found the average total cost per COPD patient ranged between CAN $2444 from a patient perspective to CAN $6693 from a societal perspective. Moreover, data suggests that the costs rise as the disease severity increases. The clinical burden review found evidence which indicates that healthcare resource utilization increases with exacerbation severity [3, 32], increasing age [46, 76], and comorbid cardiovascular disease [53]. Thus, clinicians should focus on ensuring proper diagnosis, optimizing appropriate care, and the importance of personalized medicine.

This review, like all reviews, is limited by publication bias with respect to the articles that are available. In addition, the articles in this review were a priori limited to the English language and restricted to those published since 2000 to examine the most recent data as the practice of medicine and related burden may change over time. Spatial restrictions were also applied, limiting studies to Canadian populations. However, in spite of these limitations, this review was systematic in nature and therefore by reviewing all available and relevant data, it provides a better and comprehensive understanding of the literature with respect to clinical, humanistic and economic burden of COPD in the Canadian population.

## Conclusions

COPD is currently the fourth leading cause of death among Canadians. This review found that COPD causes a profound impact on healthcare resources and produces a significant clinical, humanistic and economic burden in Canada. This review found evidence that self-care management programs, telephone support services, and integrated care programs may help limit the overall burden to Canadian patients and society.

## Abbreviations

AECOPD: acute exacerbation of chronic obstructive pulmonary disease
BMI: body mass index
BPI: Brief Pain Inventory Scale
CHAMPS: community health activities model program for seniors
COPD: chronic obstructive pulmonary disease
CRD: chronic respiratory disease
CRQ: Chronic Respiratory Questionnaire
CV: cardiovascular
ED: emergency department
GOLD: global initiative for chronic obstructive lung disease
HRQoL: health-related quality of life
IAC: inhaled anti-cholinergic
NICE: National Institute for Health and Care Excellence
PRISMA: preferred reporting items for systematic reviews and meta-analyses
PY: patient years
RCT: Randomized Controlled Trial
SF-36: short form 36
SGRQ: St George’s Respiratory Disease Questionnaire
SIP: sickness impact profile
STROBE: strengthening the reporting of observational studies in epidemiology
